# Metabolomic Profiling Reveals Brain Lipid Alterations in *PEX7*-Deficient Models of Rhizomelic Chondrodysplasia Punctata

**DOI:** 10.3390/biom16010006

**Published:** 2025-12-19

**Authors:** Riya Sankhe, Meredith I. Williams, Wedad Fallatah, Laura Mackay, Mary Layne Brown, Pranjali Bhagwat, Sarah H. Elsea, Nancy Braverman, Michael F. Wangler

**Affiliations:** 1 Department of Molecular and Human Genetics, Baylor College of Medicine (BCM), Houston, TX 77030, USA; riya.sankhe@bcm.edu (R.S.); pranjalikb@gmail.com (P.B.); sarah.elsea@bcm.edu (S.H.E.); 2 Jan and Dan Duncan Neurological Research Institute, Texas Children’s Hospital (TCH), Houston, TX 77030, USA; 3 Development, Disease Models and Therapeutics Graduate Program, Baylor College of Medicine (BCM), Houston, TX 77030, USA; meredith.williams@bcm.edu; 4 Center for Precision Environmental Health, Baylor College of Medicine (BCM), Houston, TX 77030, USA; 5 Genetics Laboratories, Department of Genetic Medicine, Kennedy Krieger Institute, Johns Hopkins University, Baltimore, MD 21287, USA; fallatah@kennedykrieger.org; 6 Department of Pediatrics, UT Southwestern Medical Center, Dallas, TX 75390, USA; laura.mackay@utsouthwestern.edu; 7 Department of Plant and Wildlife Sciences, Brigham Young University, Provo, UT 84604, USA; marymlayne@gmail.com; 8 BCM Human Genome Sequencing Center, Baylor College of Medicine (BCM), Houston, TX 77030, USA; 9 Departments of Pediatrics and Human Genetics, McGill University and the Research Institute of the McGill University Health Center, Montreal, QC H4A 3J1, Canada; nancy.braverman@mcgill.ca

**Keywords:** peroxisome, *Pex7*, metabolomics, mouse models, peroxisome biogenesis disorders, plasmalogen, ether lipids

## Abstract

Rhizomelic chondrodysplasia punctata type 1 (RCDP1) is a peroxisomal disorder characterized by skeletal shortening, intellectual disability, seizures, cataracts, and reduced lifespans. RCDP1 is caused by biallelic loss-of-function variants in *PEX7*, which encodes a protein required for importing select enzymes into the peroxisome matrix, including those essential for ether lipid synthesis (e.g., plasmalogens) and the branched-chain fatty acid catabolism. Plasmalogen deficiency is a hallmark of RCDP1 and other peroxisomal disorders, including RCDP types 2-5 (RCDP2-5) and Zellweger spectrum disorders (ZSD). Here, we performed comprehensive metabolomic profiling of clinical samples from RCDP patients and *Pex7*-deficient mouse models. We identified profound neurometabolic disturbances in the cerebral cortex and cerebellum of *Pex7*-deficient mice involving multiple lipid classes, including phosphatidylethanolamines (PEs), phosphatidylcholines (PCs), acylcarnitines, and sphingomyelins. Notably, many of these neurometabolic alterations were absent in patient and *Pex7*-deficient mouse plasma, indicating that plasma-based profiling can underrepresent the extent of CNS lipid remodeling. Overall, these findings reveal novel insights into neurometabolic adaptations to plasmalogen deficiency and suggest the potential involvement of additional pathways that may contribute to neurological dysfunction in RCDP.

## 1. Introduction

Peroxisomal biogenesis disorders (PBDs) are ultra-rare genetic diseases caused by biallelic loss-of-function (LOF) variants in *PEX* genes, which are required for peroxisome assembly, structure, replication, and metabolic function. Peroxisomes are organelles central to key metabolic processes, including fatty acid alpha- and β-oxidation, plasmalogen and bile acid biosynthesis, free radical detoxification, and regulation of cell signaling pathways [1]. PBDs encompass two major clinical groups, Zellweger spectrum disorders (ZSD) and rhizomelic chondrodysplasia punctata type 1 (RCDP1) [2]. Zellweger spectrum disorder (PBD-ZSD, OMIM #601539) results from defects in the formation of peroxisomal membranes and/or the import of all peroxisomal matrix proteins, leading to the global disruption of peroxisomal metabolic functions. RCDP1 is caused by biallelic loss-of-function variants in the *PEX7* gene required for the import of alkylglycerone phosphate synthase (AGPS) and phytanoyl-CoA hydroxylase (PHYH), enzymes, respectively, essential for ether lipid biosynthesis and branched-chain fatty acid catabolism into the peroxisome matrix [3,4]. Ether lipids, such as plasmalogens, play integral roles in membrane structure and function, act as cell signaling mediators, and possess antioxidant properties [5]. Plasmalogen deficiency occurs in a number of peroxisomal disorders, but its severity and clinical impact are most pronounced in RCDP [6]. Classic RCDP presents with proximal limb shortening, punctate bone calcifications, facial dysmorphism, congenital cataracts, severe growth retardation, and profound neurocognitive impairment [7]. Milder forms of disease associated with residual plasmalogen levels typically are characterized by growth deficiencies, developmental delays, joint contractures, cataracts, and behavioral abnormalities [6].

The neurological features of RCDP are likely to relate to the ether phospholipid defect. In PEX7-related RCDP, human and mouse data show that the severity of plasmalogen deficiency correlates with hypomyelination, cerebellar atrophy, and neurodevelopmental deficits, lesions that underlie seizures and cognitive impairment [8]. Plasmalogens are enriched in synaptic and myelin membranes and help organize lipid microdomains that position ion channels and receptors; their loss perturbs vesicle fusion and neurotransmitter release, increasing network excitability and impairing synaptic efficiency [9]. Plasmalogens act as endogenous antioxidants; their depletion increases vulnerability of neurons to oxidative injury and mitochondrial dysfunction, both linked to seizure susceptibility and impaired cognition [10]. Finally, recent reviews and translational studies reinforce that restoring plasmalogen levels can normalize synaptic signaling pathways and improve neurophysiology, further supporting a causal role for ether-lipid loss in RCDP neurological phenotypes. In summary, emerging evidence indicates that ether-lipid deficiency found in RCDP affects the membrane organization, synaptic transmission, myelination, and redox balance in the CNS.

Metabolomic and lipidomic studies demonstrate that biochemical abnormalities in PBDs extend well beyond primary peroxisomal metabolic pathways. In severe PBD-ZSD models, mitochondrial dysfunction has been observed [11,12]. Moreover, biochemical studies of plasma and cultured fibroblasts from patients with PBD-ZSD have revealed widespread defects in phospholipid metabolism [13,14,15]. Fly and mouse PBD-ZSD models exhibit altered carbohydrate homeostasis [16], and the metabolomic analysis of plasma from PBD-ZSD patients demonstrates abnormally low sphingomyelin levels [17]. Similarly, there is evidence that RCDP, which is characterized by ether-lipid biosynthesis defects as described above, exhibits alterations of downstream metabolic pathways [18]. In cultured fibroblasts from RCDP patients and brain tissue from plasmalogen-deficient mice, phosphatidylethanolamine (PE) levels were tightly regulated to those of ethanolamine plasmalogen to maintain homeostasis [19]. Moreover, *Pex7*-deficient mice showed tissue-specific elevations in C26:0-LPC levels, graded phytanic acid accumulation in plasma and cerebellar tissues (consistent with expected impaired branched-chain fatty acid catabolism), and global brain neurotransmitter deficits (Fallatah et al., 2022 [20]).

A deeper understanding of metabolomic dysfunction in RCDP and related peroxisomal disorders could inform their diagnosis, prognosis, and therapeutic development. To investigate the metabolic basis of the clinical manifestations of RCDP and other peroxisomal disorders, we performed comprehensive metabolomic profiling of plasma from a cohort of RCDP patients and tissues from an allelic series of *Pex7*-deficient mouse models [20]. We highlight key metabolic abnormalities and demonstrate the value of integrating data from model organisms and clinical samples.

## 2. Materials and Methods

### 2.1. Human Subjects

All the RCDP and ZSD human subjects were recruited to an IRB-approved research study (H-44779) at Baylor College of Medicine. All subjects consented to the research and publication.

### 2.2. Human Plasma Metabolomics Study

Plasma study was obtained from peripheral whole blood collected in EDTA-containing tubes and analyzed using the clinical Metabolon Global MAPS metabolic profiling platform (Metabolon Inc., Morrisville, NC, USA). This platform performs small molecule metabolomic profiling with clinical interpretation relevant to inborn errors of metabolism. Methodological details of this platform and the methods have been described for rare diseases [17,21,22,23,24]. Pathway analyses from a larger dataset incorporating the RCDP cases reported here were previously published [18]. The same Global MAPS platform and mass spectroscopy (MS) methods used for mouse metabolic profiling (below) were applied to human plasma samples.

### 2.3. Statistical Analysis of Human Metabolomic Data

#### 2.3.1. Data Processing and Z-Score Computation

Clinical and genomic information from patients diagnosed with RCDP and ZSD were integrated with metabolomic profiles. For each metabolite and sample, Z-scores were calculated to standardize the data, enabling comparisons between the different disorders.

#### 2.3.2. Statistical Approach: Clustering and Visualization

**Figure 1:**
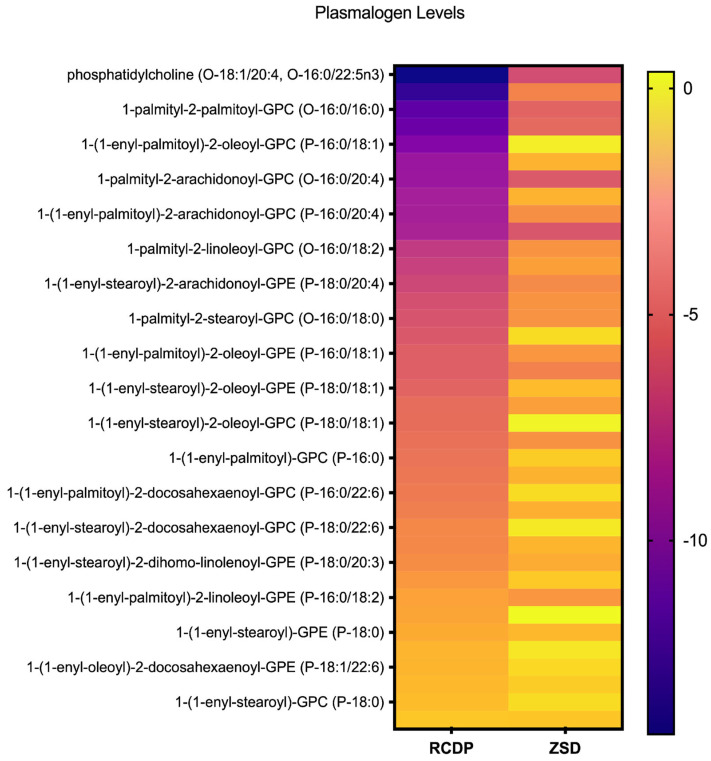
Heatmap of plasmalogen levels in rhizomelic chondrodysplasia punctata (RCDP) and Zellweger spectrum disorder (ZSD). Each row represents an individual plasmalogen species identified by molecular composition, and each column corresponds to RCDP or ZSD. Color intensity reflects relative abundance, with deeper purple indicating lower levels and brighter yellow indicating higher levels. This visualization highlights the variation in plasmalogen profiles across species and conditions, allowing for an overview of distribution patterns within the dataset.

**Figure 2:**
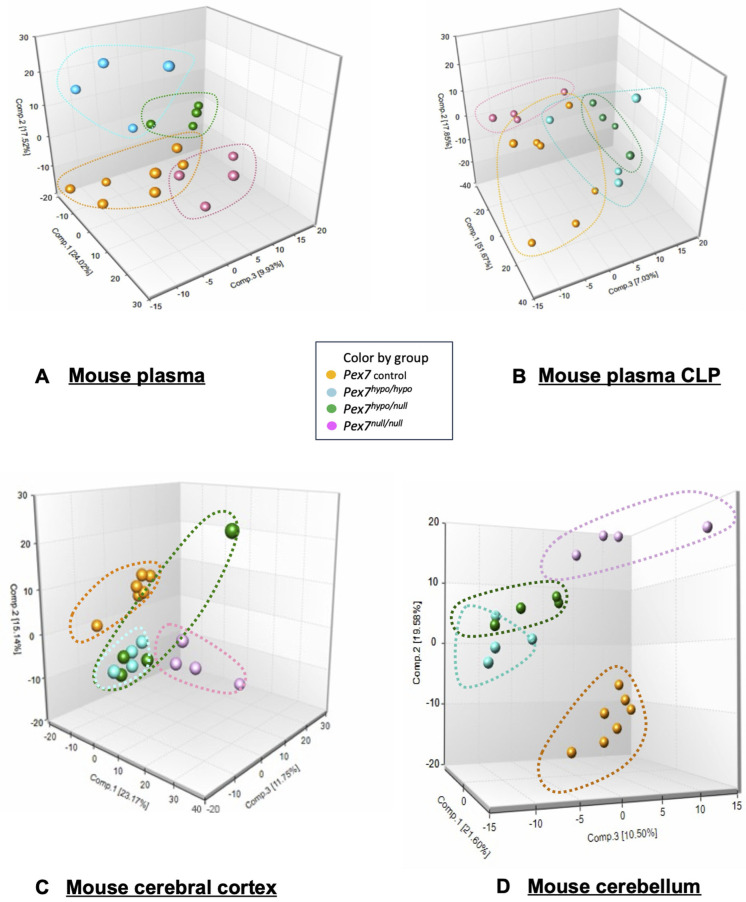
Principal component analysis (PCA) of metabolomic profiles in plasma, cerebral cortex, and cerebellum from *Pex7*-deficient mice. Data is colored-coded according to genotype *Pex7 Control* (orange), *Pex7^hypo/hypo^* (light blue), *Pex7^hypo/null^* (green), and *Pex7^null/null^* (pink/purple). (**A**) Plasma: 3D PCA plots (two different views) illustrate a clear separation of the four genotype groups, with each group forming distinct clusters outlined by dashed lines matching the respective group colors. The first three principal components explain ~51% of the total variance (PC1: ~24%, PC2: ~17%, PC3: ~10%). (**B**) Mouse plasma CLP (**C**) Cerebral cortex: PCA reveals partial separation among groups, with some overlap between *Pex7^hypo/null^* and *Pex7^null/null^*. The first three principal components explain ~49% of variance (PC1: 23%, PC2: 15%, PC3: 11%). (**D**) Cerebellum: Genotype groups form tight, well-separated clusters with the first three principal components accounting for ~51% of variance (PC1: ~21%, PC2: ~20%, PC3: ~10%).

Unsupervised clustering analysis based on the Z-score profiles was performed to evaluate the relationships between the disease groups (RCDP and ZSD). The resulting clusters were visualized using scatter plots, with each disease group represented by a different color (blue for RCDP, green for ZSD) (Figure 1 and Figure 2). These visualizations highlighted recurring patterns or disease-specific signatures. Between-group comparisons were performed using the Wilcoxon *t*-test to Z-scores.

#### 2.3.3. Sample Collection and Metabolomic Profiling of Pex7-Deficient Mice

Previously reported *Pex7*-deficient mouse strains (Fallatah et al., 2022 [20]) were used: B6;129S6-*Pex7*^tm2.0Brav^ (named here as *Pex7*^hypo/hypo^), (B6;129S6-*Pex7*^tm2.2Brav^) referred to as *Pex7*^null/null^ and (B6;129S6-*Pex7*^tm2.3Brav^) referred to as *Pex7*^hypo/null^. Plasma, cerebral cortex, and cerebellar tissues were harvested and stored at −80 °C until analysis. This study was conducted under a McGill University (Montreal, QC, Canada) animal-care-committee-approved protocol (#5538).

Metabolomic profiling for *Pex7*-deficient mouse samples was performed using the Metabolon, Inc. platform. Briefly, samples were prepared using an automated MicroLab STAR system. Proteins were removed by methanol precipitation followed by centrifugation, and each extract was divided into five fractions that were stored overnight under nitrogen until analysis. Two fractions were used for two separate reverse phase (RP) Ultrahigh Performance Liquid Chromatography–Tandem Mass Spectroscopy (UPLC-MS/MS) with positive ion mode electrospray ionization (ESI). One analysis was optimized for hydrophilic compounds and the other for hydrophobic compounds. Another fraction was used for RP/UPLC-MS/MS with negative ion mode ESI, another for analysis by HILIC/UPLC-MS/MS with negative ion mode ESI, and the remaining fraction was archived for future use. Quality control (QC) samples were processed alongside QC samples. The UPLC utilized a Waters ACQUITY ultra-performance liquid chromatography and a Thermo Scientific Z-Exactive high-resolution/accurate mass spectrometer interfaced with a heated electrospray ionization (HESI-II) source. The Orbitrap mass analyzer operated at 35,000 mass resolution. The scan range covered 70–1000 *m*/*z*.

#### 2.3.4. Metabolomic Informatics

Raw data extraction, peak detection, compound identification, and QC were performed using proprietary software from Metabolon, Inc. (Morrisville, NC, USA). Compounds were identified by comparison to internal libraries of purified standards. Identification criteria included retention time/index (RI), mass-to-charge ratio (*m*/*z*), and chromatographic data. Peak areas were quantified using area-under-the-curve analysis, and data were statistically normalized to minimize day-to-day analytical variation.

#### 2.3.5. Additional Complex Lipid Analysis

Targeted complex lipid analysis was performed using an automated butanol-methanol (BUME) extraction [25] in the presence of deuterated internal standards.

#### 2.3.6. Statistical Analysis of Mouse Metabolomic Data

For comparison between mouse groups, one-way ANOVA was used, followed by Dunnett’s multiple-comparison test where appropriate. Statistical significance was defined as *p* < 0.05.

#### 2.3.7. Principal Components Analysis (PCA)

PCA was performed to assess global metabolomic variation across mouse genotypes. It reduces data dimensionality by transforming metabolite abundances into a set of uncorrelated components, each capturing a proportion of the total variance. The percentage of variance explained by each component was calculated as the ratio of the component’s variance to the total variance.

## 3. Results

### 3.1. Plasma Metabolomic Profiling in Patients with RCDP

Plasma samples were obtained from 18 human subjects with RCDP who were recruited under an IRB approved study H-44779 at Baylor College of Medicine. Samples were collected at a family meeting organized by the RhizoKids International foundation. The age and phenotype severity of these subjects are summarized in Table 1. Two subjects (a sibling pair) were classified as nonclassic (intermediate severity), one as nonclassic (mild) [6], and the remainder exhibited classic (severe) RCDP phenotypes. Sixteen of 18 subjects had pathogenic variants in *PEX7* (RCDP1), while the sibling pair harbored biallelic deleterious loss-of-function (LOF) variants in *GNPAT* [26] (RCDP2). Participant ages ranged from 9 months to 16 years. The analysis also included three previously reported individuals with ZSD [17]. This cohort comprised mild and intermediate but not severe ZSD unrelated to peroxisome function (Table S1). In total, 941 named biochemicals were quantified among 1177 molecules, including unknowns. For each compound and each sample, a Z-score was assigned (Table S2).

### 3.2. Severe Ether Lipid Deficiencies in RCDP

Compounds with an average Z-score < −2 across all the RCDP samples were classified as ‘decreased’, resulting in a total of 43 decreased compounds, including several unknowns. Of the 43 decreased compounds in RCDP plasma, 34 were identified as etherlipids, including plasmanyl ether lipids, plasmalogens, or lysoplasmalogens (Table S3). In fact, the 12 most profoundly reduced compounds were all ether lipids.

Among the 38 ether lipid species profiled (Figure 1), most exhibited substantial reductions in RCDP samples. For example, 1-palmityl-2-oleoyl-GPC (O-16:0/18:1) exhibited an average Z-score of −13.3, while 1-palmitoyl phosphatidylcholine (O-18:1/20:4, O-16:0/22:5n3) had an average Z-score of −14.27 in RCDP samples (Figure S1A,B). Only four of the 38 ether lipid species had average levels within the normal range in RCDP samples (Figure 1). Most of the ether lipid species measured were also reduced in mild or mild-intermediate ZSD samples; however, the magnitude of the Z-score difference was substantially less pronounced in ZSD. Notably, some ether lipid species were normal in ZSD but were reduced in RCDP, such as the plasmalogen, 1-(1-enyl-palmitoyl)-2-palmitoyl-GPC (P-16:0/16:0) (Figure S1C). Overall, ZSD and RCDP samples exhibited multiple ether lipids, including plasmalogen species with markedly decreased levels; however, these reductions were far more extensive and severe in RCDP compared to the ZSD samples, with a greater number of species affected.

### 3.3. Distinct Sphingomyelin Profiles in ZSD and RCDP

**Figure 3:**
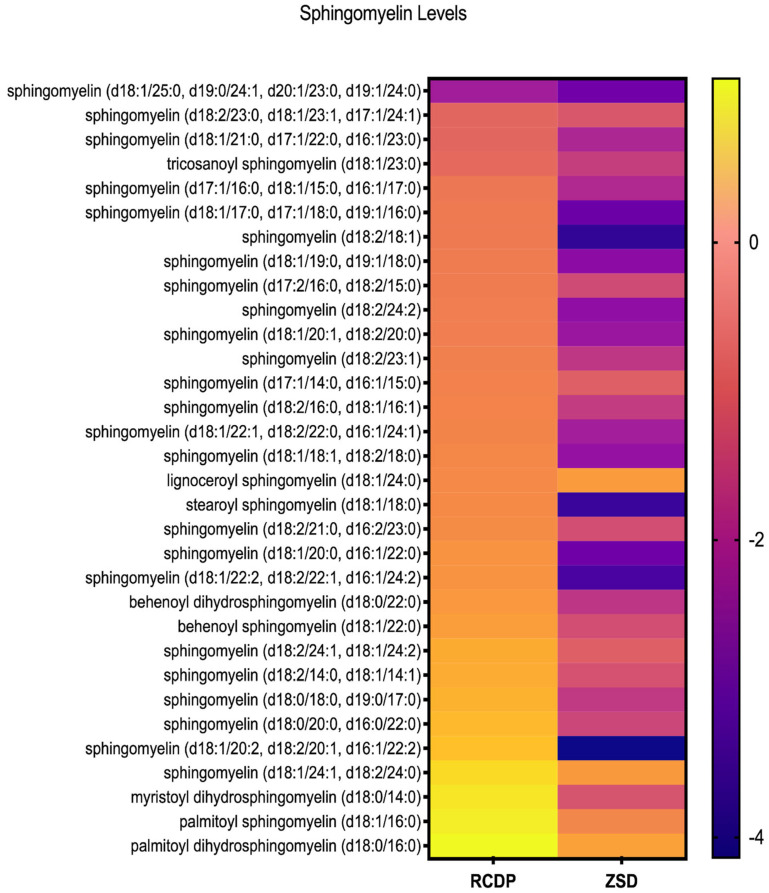
Heatmap of sphingomyelin levels in RCDP and ZSD patient-derived plasma. Each row represents a distinct sphingomyelin species, and each column corresponds to the genetic condition. Color scale reflects relative abundance, with purple indicating lower levels and yellow indicating higher levels. The heatmap reveals consistently lower plasma sphingomyelin levels in ZSD compared to RCDP patients, whose plasma sphingomyelin levels show intermediate abundance.

We previously reported reductions in multiple sphingomyelin species in ZSD plasma [17]. Here, we studied our cohort of RCDP plasma and included 3 ZSD samples for comparison. We noted that the nominal reductions in sphingomyelin were substantially less pronounced in RCDP relative to ZSD (Figure 3). Because we compared only 3 ZSD samples, we did not observe statistically significant differences (Figure S2).

### 3.4. Global Metabolomic Profiling in Plasma and Brain Tissues from Pex7-Deficient Mouse Models

To investigate the impact of RCDP on brain metabolism, we analyzed a series of *Pex7*-deficient mouse models (Table S4). Plasma, cerebral cortex, and cerebellar tissues were collected from the following genotypes: (1) *Pex7^WT/WT^* (littermate control), (2) *Pex7^hypo/hypo^*, (3) *Pex7^hypo/null^*, and (4) *Pex7^null/null^*. These previously characterized strains span the severity spectrum observed in human RCDP and exhibit a clear genotype-phenotype correlation, with increasing plasmalogen deficiency, other biochemical abnormalities, and neurobehavioral impairments across genotypes (*Pex7^hypo/hypo^* < *Pex7^hypo/null^* < *Pex7^null/null^*) [20]. In addition, comprehensive lipid profiling (CLP) was performed on plasma samples from these mice.

### 3.5. Metabolomic Profiling Distinguishes Severity of Pex7 Loss of Function in RCDP Mouse Models

Principal components analysis (PCA) of plasma metabolomic data revealed distinct clustering among control, *Pex7^hypo/hypo^*, *Pex7^null/null^*, and *Pex7^hypo/null^* mice (Figure 2A). PCA of the CLP data also demonstrated separation between the groups, although some overlap remained (Figure 2B). Interestingly, similar clustering by genotype was observed for the cerebral cortex (Figure 2C) and cerebellum (Figure 2D). Taken together, the PCA suggested clear metabolomic and lipidomic differences between control and *Pex7*-deficient mice, consistent with genotype-phenotype correlations across both platforms. Metabolon also analyzed plasma using a targeted Complex Lipid Panel (CLP), which isolates and quantifies specific phospholipid and sphingolipid subclasses using targeted mass spectrometry. The CLP data revealed clear genotype-dependent shifts across multiple lipid classes, further supporting the metabolic severity gradient observed in the PCA results.

### 3.6. Pex7-Deficient Mouse Models Exhibit Brain-Specific Lipid Abnormalities Undetected in Plasma

We compared metabolomic profiles across plasma, cerebral cortex, and cerebellar tissues from *Pex7*-deficient mice. Plasmalogens were markedly decreased in mouse plasma (Figures S3 and S4), consistent with our prior findings in human plasma. Similar reductions were observed in the cerebral cortex and cerebellum of *Pex7*-deficient mice, suggesting a global reduction in plasmalogens in plasma and brain tissue.

**Figure 4:**
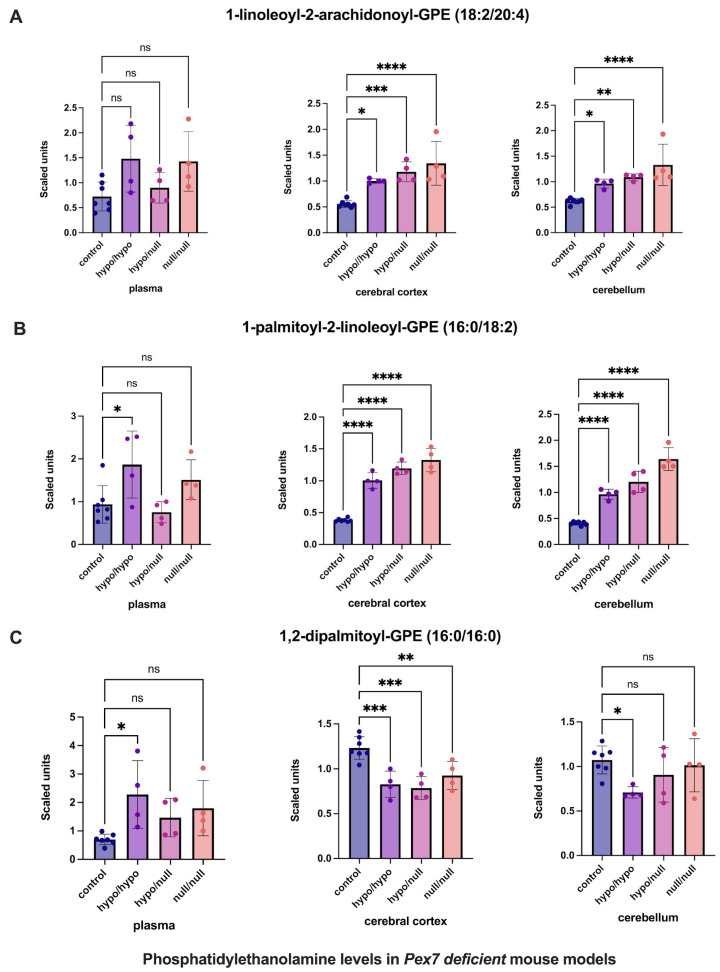
Altered phosphatidylethanolamine (PE) levels in *Pex7*-deficient mouse models. Panels show three representative PE species: (**A**) 1-linoleoyl-2-arachidonoyl-GPE, (**B**) 1-palmitoyl-2-linoleoyl-GPE, and (**C**) 1,2-dipalmitoyl-GPE. Both cerebral cortex (**A**) and cerebellum (**B**) exhibit significant, genotype-dependent elevations across mutant groups (*p* < 0.05 for most comparisons). In contrast, (**C**) shows a unique reduction in the cerebral cortex of *Pex7*-deficient mice while remaining unchanged in plasma and cerebellum. Plasma levels of all three species are generally unaffected or show only modest, non-significant differences. Collectively, this highlights brain-specific PE metabolic adaptations in *Pex7*-deficient mice that are not reflected in plasma profiles. *p*-values as follows: ns: not significant, * *p* < 0.05, ** *p* < 0.01, *** *p* < 0.001, **** *p* < 0.0001.

For phosphatidylethanolamines (PEs) (Figure 4), previous studies suggested compensatory elevations in tissue, but not plasma, in response to plasmalogen deficiency [19]. Consistent with this, several PEs were increased in the cerebral cortex and cerebellum while remaining unchanged in mouse plasma (Figure 4A,B). However, 1,2-dipalmitoyl-GPE (16:0/16:0) exhibited decreased levels in the cerebral cortex, with normal levels in cerebellum and plasma (Figure 4C).

**Figure 5:**
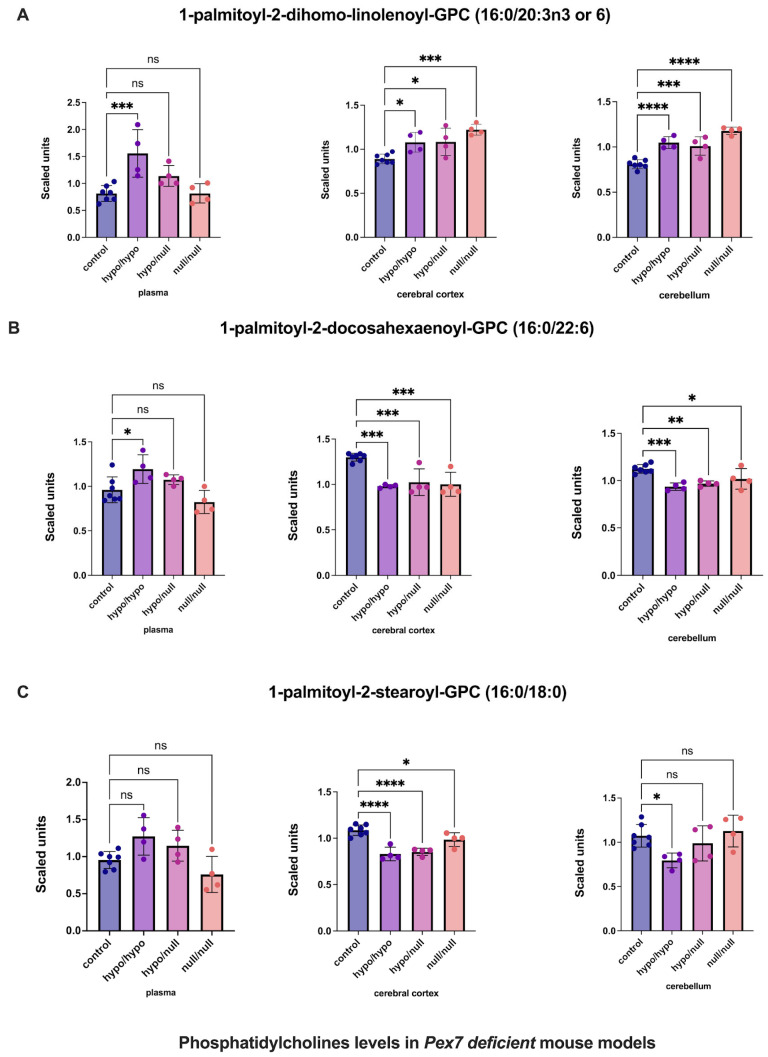
Altered phosphatidylcholine (PC) levels in *Pex7*-deficient mouse models. Panels show three representative phosphatidylcholine (PC) species: (**A**) 1-palmitoyl-2-dihomo-linolenoyl-GPC, (**B**) 1-palmitoyl-2-docosahexaenoyl-GPC, and (**C**) 1-palmitoyl-2-stearoyl-GPC. Across Pex7-deficient genotypes, (**A**,**B**) exhibit significant elevations in the cerebral cortex and cerebellum (*p* < 0.05 for most comparisons), while plasma levels show only minor or nonsignificant changes. For (**C**), cerebral cortex levels are significantly increased, whereas cerebellar and plasma levels remain largely unchanged. Together, these data reveal brain-specific differences in PC metabolism in *Pex7*-deficient mice that are not consistently reflected in the corresponding plasma metabolomic profiles. *p*-values as follows: ns: not significant, * *p* < 0.05, ** *p* < 0.01, *** *p* < 0.001, **** *p* < 0.0001.

Multiple phosphatidylcholines (PCs) exhibited complex tissue-specific alterations (Figure 5 and Figure S5). For 1-palmitoyl-2-dihomo-linolenoyl-GPC (16:0/20:3n3 or 6), plasma showed nominal, nonsignificant increases, whereas cerebral cortex and cerebellum demonstrated significant elevations (Figure 5A). Similarly, 1-palmitoyl-2-docosahexaenoyl-GPC (16:0/22:6) displayed nonsignificant plasma changes, whereas the cerebral cortex and cerebellum showed clear, significant increases (Figure 5B). In contrast, 1-palmitoyl-2-stearoyl-GPC (16:0/18:0) showed no plasma abnormalities but significant elevations in both brain regions, with the cerebral cortex demonstrating the largest effect size (Figure 5C).

Sphingomyelin profiles showed distinct trends between human plasma and *Pex7*-deficient mice (Figure S6). Palmitoyl sphingomyelin (d18:1/16:0) was significantly elevated (Z-score > 2) in humans and increased in two of the three mutant mouse genotypes. Stearoyl sphingomyelin (d18:1/18:0) showed no significant change in humans (Z-score between 2 and −2), but was significantly elevated across all *Pex7*-deficient mouse genotypes. Similarly, sphingomyelins (d17:1/16:0, d18:1/15:0, d16:1/17:0) were significantly elevated in both human and all *Pex7*-deficient mice (Figure S6). Consistent with these brain-specific lipid alterations, acylcarnitines also exhibited alterations in the cerebral cortex and cerebellum but remained unchanged in plasma in both humans and *Pex7*-deficient mice (Figure S7).

**Figure 6:**
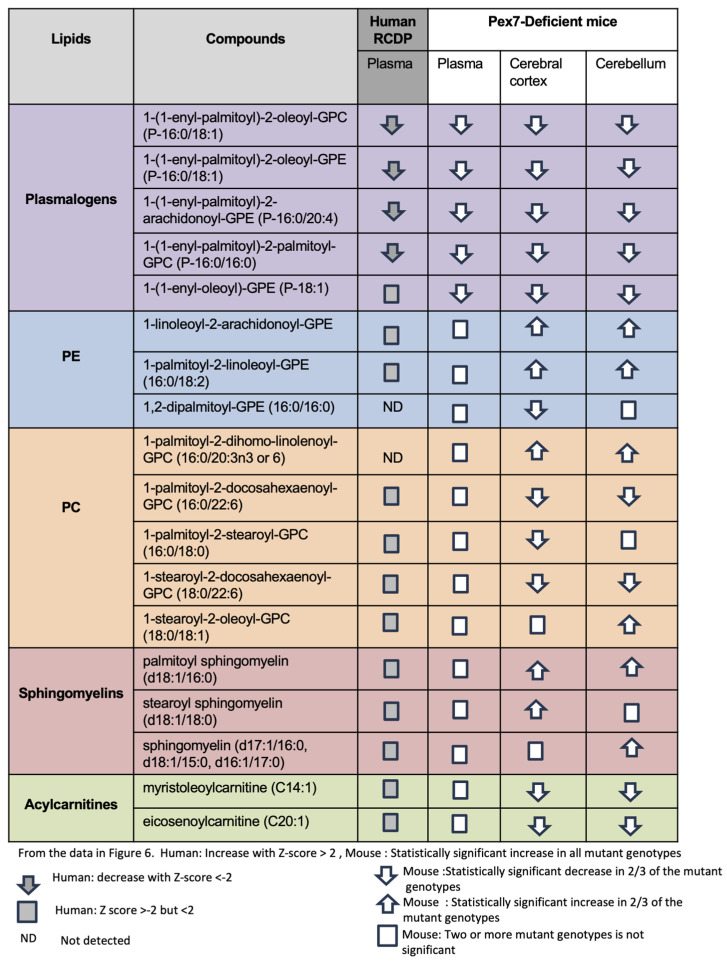
Integrated comparative analysis of lipid metabolite changes across RCDP patient-derived plasma and *Pex7*-deficient mouse plasma and brain tissue. Lipid species are grouped into five major classes: plasmalogens, phosphatidylethanolamines (PE), phosphatidylcholines (PC), sphingomyelins, and acylcarnitines. Each row represents an individual lipid species, and columns correspond to sample types: human RCDP plasma, mouse plasma, mouse cerebral cortex, and mouse cerebellum. Directional arrows indicate changes in metabolite levels based on Z-scores and statistical significance). For human samples, an upward arrow shows a significant increase (Z-score > 2), a downward arrow shows a significant decrease (Z-score < −2), and a square represents a small or borderline change (−2 ≤ Z ≤ 2). “ND” indicates a lipid that was not detected. For mouse samples, an upward or downward arrow indicates a consistent increase or decrease observed in at least two out of three Pex7 mutant genotypes (considered significant). A square indicates that the change was not consistent across mutants and is therefore not significant. Statistical thresholds: human samples show significant changes with |Z| > 2; for mouse data, significance reflects a consistent effect in two-thirds of mutant genotypes.

We summarized changes across major lipid classes (including plasmalogens, PEs, PCs, sphingomyelins, and acylcarnitines) by comparing representative compounds in human plasma samples and *Pex7*-deficient mouse models (Figure 6). Plasmalogen deficiency was nearly uniform across datasets (Figure 6, purple rows). Among PEs, species such as 1-palmitoyl-2-linoleoyl-GPE were normal in human and mouse plasma but exhibited large increases in cerebellum and cerebral cortex, consistent with prior reports [19]. However, 1,2 dipalmitoyl-GPE (16:0/16:0) is decreased in the mouse cerebral cortex, indicating that not all PEs follow the same trend (Figure 6, blue rows). For PCs, the effects were highly complex. While *Pex7*-deficient mouse or human patient plasma were unaltered, some PCs were increased in the cerebral cortex and cerebellum, some PCs were increased in both cerebral cortex and cerebellum, others were decreased, and several were altered exclusively in either the cerebral cortex or cerebellum (Figure 6, orange rows).

Sphingomyelins previously noted to show a reduction in PBD-ZSD plasma samples showed minimal plasma alterations in both RCDP and *Pex7*-deficient mice. However, several sphingomyelin species were elevated in the cerebral cortex, cerebellum, or both (Figure 6, salmon rows). Similarly, while plasma acylcarnitines were typically normal, both cerebral cortex and cerebellum exhibited increased levels (Figure 6, green rows).

Overall, *Pex7*-deficient mice exhibited consistent plasmalogen depletion across plasma and brain tissues alongside complex alterations in PEs, PCs, sphingomyelins, and acylcarnitines. Notably, several lipid abnormalities were detected exclusively in the cerebral cortex and cerebellum, underscoring brain-specific metabolic changes not evident in plasma.

## 4. Discussion

RCDP is a peroxisomal disorder, most commonly caused by biallelic pathogenic variants in *PEX7* and characterized by severe skeletal, neurological, and developmental abnormalities [3,27]. RCDP exhibits clinical findings and a natural history distinct from other peroxisomal disorders [28] and is defined biochemically by ether lipid and plasmalogen deficiency as its most hallmark feature [29]. Nevertheless, the molecular mechanisms underlying the profound neurocognitive impairment in RCDP remain incompletely understood.

Here, we have applied global metabolomic profiling to plasma samples from RCDP patients and plasma and brain specimens from a series of *Pex7*-deficient mice. A key finding is that sphingomyelin reductions, previously reported for ZSD [17], were largely absent in both RCDP patient plasma and *Pex7*-deficient mouse plasma. While global peroxisomal dysfunction drives plasma sphingomyelin depletion in ZSD, RCDP exhibits selective plasmalogen loss (and impaired phytanic acid catabolism for RCDP1) with the relative preservation of plasma sphingomyelin. This analysis was limited by the relatively small number of samples, such that our comparisons include three individuals with ZSD, although we have previously analyzed 18 samples from ZSD with similar results (Wangler et al., 2018 [17]). We do see a consistent pattern between the results for these individuals and our previously published metabolomic study [17]. Importantly, we have focused our analyses of ZSD samples on mild and intermediate severity. Metabolomic profiling of *Pex7*-deficient mice revealed extensive, unexpected alterations in brain lipids, including elevated PEs, mixed increases and decreases in PCs, and selective elevations in sphingomyelin and acylcarnitine species in the cerebral cortex and cerebellum (Figure 6). By integrating data across Figure 4, Figure 5 and Figure 6, it was revealed that systemic plasmalogen depletion is consistent across plasma and brain tissues and that extensive remodeling of PEs, PCs, sphingomyelins, and acylcarnitines occurs within the brain, but not plasma. This suggests the existence of CNS-specific lipidomic changes in RCDP that are undetected by plasma-based metabolomic approaches.

Although traditional plasma biochemical testing remains essential for RCDP diagnosis, it detects plasmalogen deficiency, and the comprehensive metabolomic profiling indicated minimal changes to other analytes. Our findings in *Pex7*-deficient mice suggest that the brains of patients with RCDP may harbor numerous biochemical alterations beyond plasmalogen reductions, including dysregulation of PEs, PCs, sphingomyelins, and acylcarnitines. This underscores the potential limitations of plasma-based biomarkers to capture neurochemical changes in RCDP and likely other inherited metabolic disorders.

While the *Pex7*-deficient mouse model series is well-established and recapitulates many aspects of RCDP, species-specific differences exist. For example, the *Pex7*-deficient mice exhibit plasma C26-lysoPC alterations that are absent in patient plasma [20]. These differences highlight the value of integrating data from patient-derived specimens and model systems to elucidate disease mechanisms.

Elevated tissue PEs have been proposed as a compensatory response to plasmalogen deficiency [19]. Consistent with this, we observed increased brain PEs along with selective increases in PCs, sphingomyelins, and acylcarnitines in the *Pex7*-deficient mouse series. These may reflect adaptive metabolic changes in response to plasmalogen deficiency. However, our data also reveal additional complexity, given that certain PE and PC species were decreased in the *Pex7*-deficient mouse brain and appeared within the normal range in their plasma. This discrepancy suggests potential alterations in lipid transport or regional metabolism within Pex7-deficient murine brain tissues. Moreover, the dissociations between *Pex7*-deficient plasma and brain lipid profiles suggest constraints on the utility of plasma metabolomics for monitoring neurological disease in RCDP.

The brain-specific lipid alterations observed in *Pex7*-deficient mice provide important clues to the mechanisms underlying RCDP neuropathology. They could also represent compensatory responses to plasmalogen deficiency, as previously proposed (Dorninger et al., 2015 [19]). Given the critical roles of these lipids in membrane integrity, myelination, and synaptic signaling, these alterations may contribute directly to the neuropathology of RCDP. They also may reflect the secondary consequences of disrupted energy metabolism, such as altered acylcarnitines linked to mitochondrial dysfunction. Additional multi-cohort studies integrating lipidomic profiling with functional assays in human-derived models, including cultured cells, organoids, and assembloids, and animal models, will be needed to distinguish adaptive from pathogenic effects.

Given the roles of plasmalogens, PEs, PCs, and sphingomyelins in membrane architecture, myelin maintenance, and synaptic vesicle dynamics, the brain-specific lipid disruptions in *Pex7*-deficient mice warrant further study into their contribution to the neurodevelopmental and cognitive deficits observed in RCDP. These findings suggest that therapeutic strategies focused on restoring plasmalogens may impact phospholipid and sphingolipid homeostasis. While plasmalogen replacement therapies have been explored in *Pex7*-deficient mouse models [30], their efficacy in correcting brain pathology remains uncertain. Our findings identify novel candidate brain-specific lipid biomarkers that could inform future preclinical research and help evaluate plasmalogen replacement therapies aimed at restoring neurological functions in RCDP [30].

## 5. Conclusions

This study provides novel observations regarding metabolomic changes in RCDP. Specifically, we catalog changes in the mouse brain and cerebellum that involve PE, PC, and sphingolipids that have not been previously examined. The results suggest that RCDP can involve additional phospholipid defects that are tissue-dependent and may not be detected in plasma testing.

## Figures and Tables

**Table 1:** Characteristics of the clinical plasma samples for RCDP.

Subject ID	Affected Gene	Age at Sample Collection	RCDP Phenotypic Severity
PEX_Family29-1	*GNPAT (RCDP2)*	15 years, 1 months	Nonclassic (Intermediate)
PEX_Family29-4	*GNPAT (RCDP2)*	11 years, 8 months	Nonclassic (Intermediate)
PEX_Family28-1	*PEX7 (RCDP1)*	16 years, 0 months	Classic
PEX_Family30-1	*PEX7*	7 years, 6 months	Nonclassic (Mild)
PEX_Family31-1	*PEX7*	1 year, 4 months	Classic
PEX_Family32-1	*PEX7*	0 years, 9 months	Classic
PEX_Family33-1	*PEX7*	0 years, 11 months	Classic
PEX_Family34-1	*PEX7*	1 year, 3 months	Classic
PEX_Family35-1	*PEX7*	1 year, 0 months	Classic
PEX_Family36-1	*PEX7*	1 year, 8 months	Classic
PEX_Family37-1	*PEX7*	1 year, 0 months	Classic
PEX_Family38-1	*PEX7*	10 years, 7 months	Classic
PEX_Family39-1	*PEX7*	5 years, 11 months	Classic
PEX_Family40-1	*PEX7*	4 years, 10 months	Classic
PEX_Family41-1	*PEX7*	2 years, 3 months	Classic
PEX_Family42-1	*PEX7*	2 years, 1 month	Classic
PEX_Family43-1	*PEX7*	3 years, 10 months	Classic
PEX_Family44-1	*PEX7*	0 years, 11 months	Classic

## Data Availability

The data supporting the findings of this study are available within the article and its Supplementary Information Files.

## Supplementary Materials

The following supporting information can be downloaded at: https://www.mdpi.com/article/10.3390/biom16010006/s1, Figure S1: Z-score distribution of selected glycerophosphocholine (GPC) lipid species in plasma from individuals with RCDP and ZSD; Figure S2: Z-score distribution of individual sphingomyelin species in plasma from individuals with RCDP and ZSD; Figure S3: Marked reduction of representative plasmalogen species across plasma, cerebral cortex, and cerebellum in *Pex*7-deficient mouse models; Figure S4: Severe reduction of specific plasmalogen species across plasma and brain tissues in *Pex*7-deficient mouse models; Figure S5: Tissue-specific alterations in phosphatidylcholine (PC) species in *Pex*7-deficient mouse models; Figure S6: Altered sphingomyelin metabolism in *Pex*7-deficient mouse models; Figure S7: Altered acylcarnitine metabolism in *Pex*7-deficient mouse models; Table S1: Composition of human study cohort; Table S2: Z-scores for sphingomyelin levels in sample; Table S3: Decreased in RCDP; Table S4: Composition of *Pex*7-deficient mouse cohort.
