# Supplementary material for: Metabolomic Profiling Reveals Brain Lipid Alterations in PEX7-Deficient Models of Rhizomelic Chondrodysplasia Punctata
Source: Biomolecules. 2025 Dec 19;16(1):6. doi: 10.3390/biom16010006 (PMC12839017; doi:10.3390/biom16010006)
Supplement: Supplementary file 1 [file biomolecules-16-00006-s001.zip › Supplemental figure legends.pdf]

## Supplemental figure legends

**Table S1: Composition of human study cohort.** Summary of plasma samples collected from individuals diagnosed with peroxisome biogenesis disorders (PBDs) and other types of rhizomelic chondrodysplasia punctata (RCDP). The cohort group includes patients with RDCP (N=18) and Zellweger spectrum disorders (N=3).

**Table S4: Composition of *Pex7*-deficient mouse cohort.** Summary of sample counts collected from four *Pex7* genotypes: wild-type controls (N = 7), hypomorph/hypomorph (N = 4), hypomorph/null (N = 4), and null/null (N = 4). Sample types include plasma, cerebral cortex, and cerebellum.

**Figure S1**. **Z-score distribution of selected glycerophosphocholine (GPC) lipid species in plasma from individuals with RCDP and ZSD.** Panels (A–F) display Z-scores for six representative ether- and plasmalogen-linked GPC species in individuals with RCDP (blue circles) and ZSD (orange squares). Horizontal bars indicate group means, and

p-values from pairwise comparisons are shown above connecting lines. Panels: (A**)** 1-palmitoyl-2-oleoyl-GPC (O-16:0/18:1): RCDP vs ZSD: $p < 0.0001$. (B) Phosphatidylcholine (O-18:1/20:4, O-16:0/22:5n3): RCDP vs ZSD: $p < 0.0001$. (C) 1-(1-enyl-palmitoyl)-2-palmitoyl-GPC (P-16:0/16:0): RCDP vs ZSD: $p < 0.0001$. (D) 1-stearoyl-2-docosapentaenoyl-GPC (O-18:0/22:5n3): RCDP vs ZSD: $p = 0.0122$. (E) 1-(1-enyl-stearoyl)-2-linoleoyl-GPC (P-16:0/18:1): RCDP vs ZSD: $p = 0.0001$. (F) 1-(1-enyl-palmitoyl)-2-oleoyl-GPC (P-16:0/18:1): RCDP vs ZSD: $p < 0.0001$ Ether- and plasmalogen-GPC species are significantly decreased in RCDP relative to ZSD supporting their potential utility as diagnostic biomarkers for RCDP.

**Figure S2**. Z-score distribution of individual sphingomyelin species in plasma from individuals with RCDP and ZSD. Panels (A–F) show Z-scores of six representative sphingomyelin species measured in plasma from individuals with RCDP (blue circles) and ZSD (orange squares). Horizontal bars indicate group means, and p-values for pairwise comparisons are shown above connecting lines. (A) Sphingomyelin (d18:1/17:0, d17:1/18:0, d19:1/16:0): RCDP vs ZSD:

p = 0.1165. (B) Sphingomyelin (d18:1/20:0, d16/22:0): RCDP vs ZSD: p = 0.0813.

(C) Sphingomyelin (d18:2/18:1): RCDP vs ZSD: p = 0.0531. (D) Sphingomyelin (d18:1/22:2, d18:2/22:1, d16:1/24:2) : RCDP vs ZSD: p = 0.1165. (E) Sphingomyelin (d18:1/25:0, d19:0/24:1, d20:1/23:0, d19:1/24:0): RCDP vs ZSD: p = 0.1584. (F) Tricosanoyl sphingomyelin (d18:1/23:0): RCDP vs ZSD: p = 0.2433 While most sphingomyelin species show no statistically significant differences between RCDP and ZSD, longer-chain species (panels E and F) exhibit modest, genotype-associated alterations, suggesting subtle lipidomic differences potentially linked to peroxisomal dysfunction.

**Figure S3**. Marked reduction of representative plasmalogen species across plasma, cerebral cortex, and cerebellum in *Pex7*-deficient mouse models. Panels show levels of three representative plasmalogen species: (A) 1-(1-enyl-palmitoyl)-2-oleoyl-GPC, (B) 1-(1-enyl-palmitoyl)-2-oleoyl-GPE, and (C) 1-(1-enyl-palmitoyl)-2-arachidonoyl-GPE, measured in plasma,

cerebral cortex, and cerebellum from wild-type controls and *Pex7* mutant mice (*hypo/hypo, hypo/null, null/null*).

Across all three plasmalogen species, mutant mice exhibited robust, genotype-dependent reductions compared to controls ($p$ < 0.0001 for nearly all comparisons). The most severe depletion occurred in *Pex7-null/null* mice, with intermediate reductions in *hypo/null* and *hypo/hypo* genotypes. This pattern was consistent across plasma, cortex, and cerebellum, demonstrating that plasmalogen loss is a systemic biochemical hallmark of *Pex7*-deficiency in these models.

**Figure S4**. Severe reduction of specific plasmalogen species across plasma and brain tissues in *Pex7*-deficient mouse models. Panels show levels of two representative plasmalogen species: (D) 1-(1-enyl-palmitoyl)-2-palmitoyl-GPC and(E) 1-(1-enyl-oleoyl)-GPE, measured in plasma, cerebral cortex, and cerebellum from wild-type controls and *Pex7* mutant mice (*hypo/hypo, hypo/null, null/null*). For both species, mutant mice exhibited pronounced, genotype-dependent reductions compared to controls ($p$ < 0.0001 for nearly all comparisons). The most severe depletion occurred in *Pex7*<sup>null/null</sup> mice, with

intermediate reductions in *hypo/null* and *hypo/hypo* genotypes. These findings confirm that plasmalogen loss is a robust and consistent biochemical feature of *Pex7*-deficiency across both plasma and brain tissues in the mouse models studied.

**Figure S5.** Tissue-specific alterations in phosphatidylcholine (PC) species in *Pex7*-deficient mouse models. Panels show levels of two representative PC species measured in plasma, cerebral cortex, and cerebellum from wild-type controls and *Pex7*-deficient mice (hypo/hypo, hypo/null, null/null): (A) 1-stearoyl-2-docosahexaenoyl-GPC (18:0/22:6) and (B) 1-stearoyl-2-oleoyl-GPC (18:0/18:1). For species (A), *Pex7*-deficient mice exhibit significant decreases in cerebral cortex and cerebellum ($p < 0.0001$ for most comparisons), whereas plasma levels remain largely unchanged. For species (B), *Pex7*-deficient mouse brains show modest, genotype-dependent increases, particularly in the cerebellum, while plasma levels are variable and less consistent.

**Figure S6.** Altered sphingomyelin metabolism in *Pex7*-deficient mouse models. Panels show levels of three sphingomyelin species measured in

plasma, cerebral cortex, and cerebellum from wild-type controls and *Pex7* mutant mice (*hypo/hypo, hypo/null, null/null*): (A) Palmitoyl sphingomyelin (d18:1/16:0), (B) Stearoyl sphingomyelin (d18:1/18:0), and (C) Complex sphingomyelin species (d17:1/16:0, d18:1/15:0, d16:1/17:0). For species (A) and (B), *Pex7*-deficient mice exhibit significant increases in cerebral cortex and cerebellum ($p < 0.001$ for most comparisons), while plasma levels remain variable or unchanged. For species (C), significant increases are observed in cerebellum, with subtler or inconsistent effects in cortex and plasma.

**Figure S7. Altered acylcarnitine metabolism in *Pex7*-deficient mouse models.** Panels show levels of two representative acylcarnitine species measured in plasma, cerebral cortex, and cerebellum from wild-type controls and *Pex7* mutant mice (*hypo/hypo, hypo/null, null/null*):(A) Myristoleoylcarnitine (C14:1) and (B) Eicosenoylcarnitine (C20:1). For both species, *Pex7*-deficient mice exhibit significant reductions in cerebral cortex and cerebellum ($p < 0.01$ for most comparisons), while plasma levels remain unchanged across genotypes. The reduction is

particularly pronounced for myristoleoylcarnitine, suggesting a stronger impact on short- and medium-chain acylcarnitine metabolism.
