# Supplementary material for: Metabolomic Profiling Reveals Brain Lipid Alterations in PEX7-Deficient Models of Rhizomelic Chondrodysplasia Punctata
Source: Biomolecules. 2025 Dec 19;16(1):6. doi: 10.3390/biom16010006 (PMC12839017; doi:10.3390/biom16010006)
Supplement: Supplementary file 1 [file biomolecules-16-00006-s001.zip › Table S1.pdf]

Table S1

Human Study

**Diagnosis**	**Number of Plasma Samples**
Peroxisome Biogenesis Disorders-<br>Rhizomelic Chondrodysplasia Punctata (RCDP) (*PEX7)*	N=18
Peroxisome Biogenesis Disorders- Zellweger Spectrum Disorders (*PEX1)*	N=3
