# Supplementary figures and images for: Metabolomic Profiling Reveals Brain Lipid Alterations in PEX7-Deficient Models of Rhizomelic Chondrodysplasia Punctata
Source: Biomolecules. 2025 Dec 19;16(1):6. doi: 10.3390/biom16010006 (PMC12839017; doi:10.3390/biom16010006)

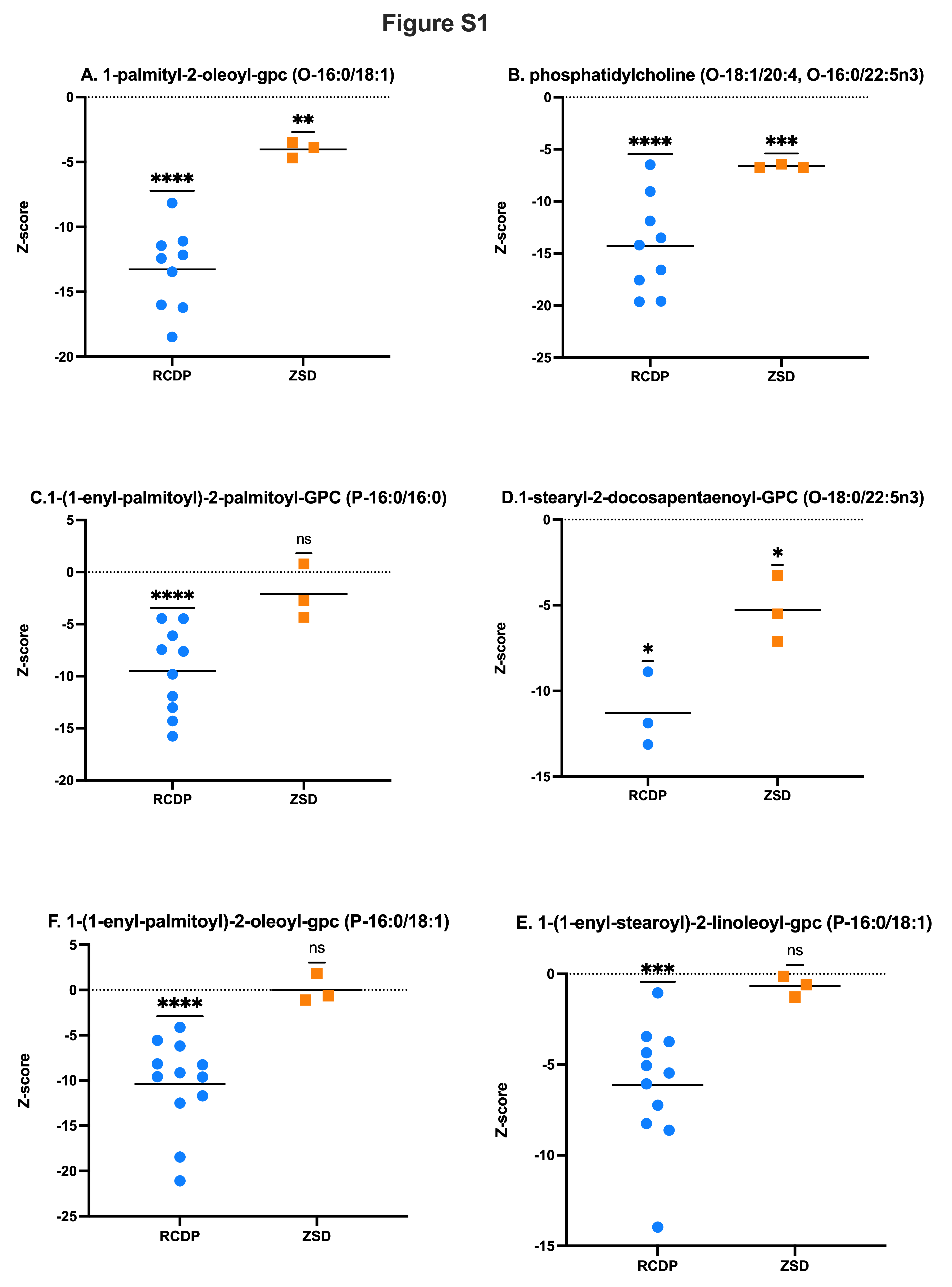

Supplement: Supplementary file 1 [file biomolecules-16-00006-s001.zip › FigureS1.tiff]

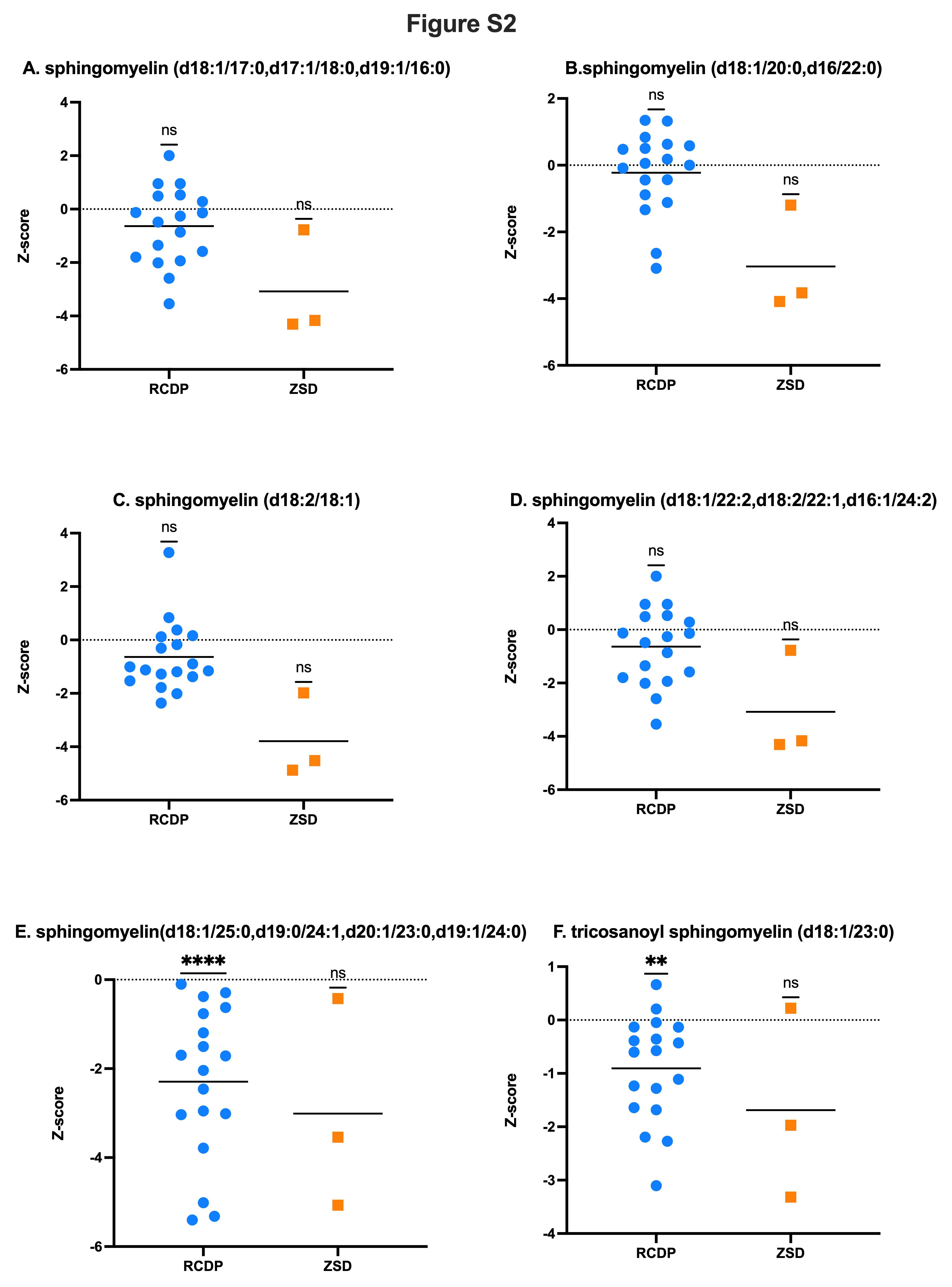

Supplement: Supplementary file 1 [file biomolecules-16-00006-s001.zip › FigureS2.tiff]

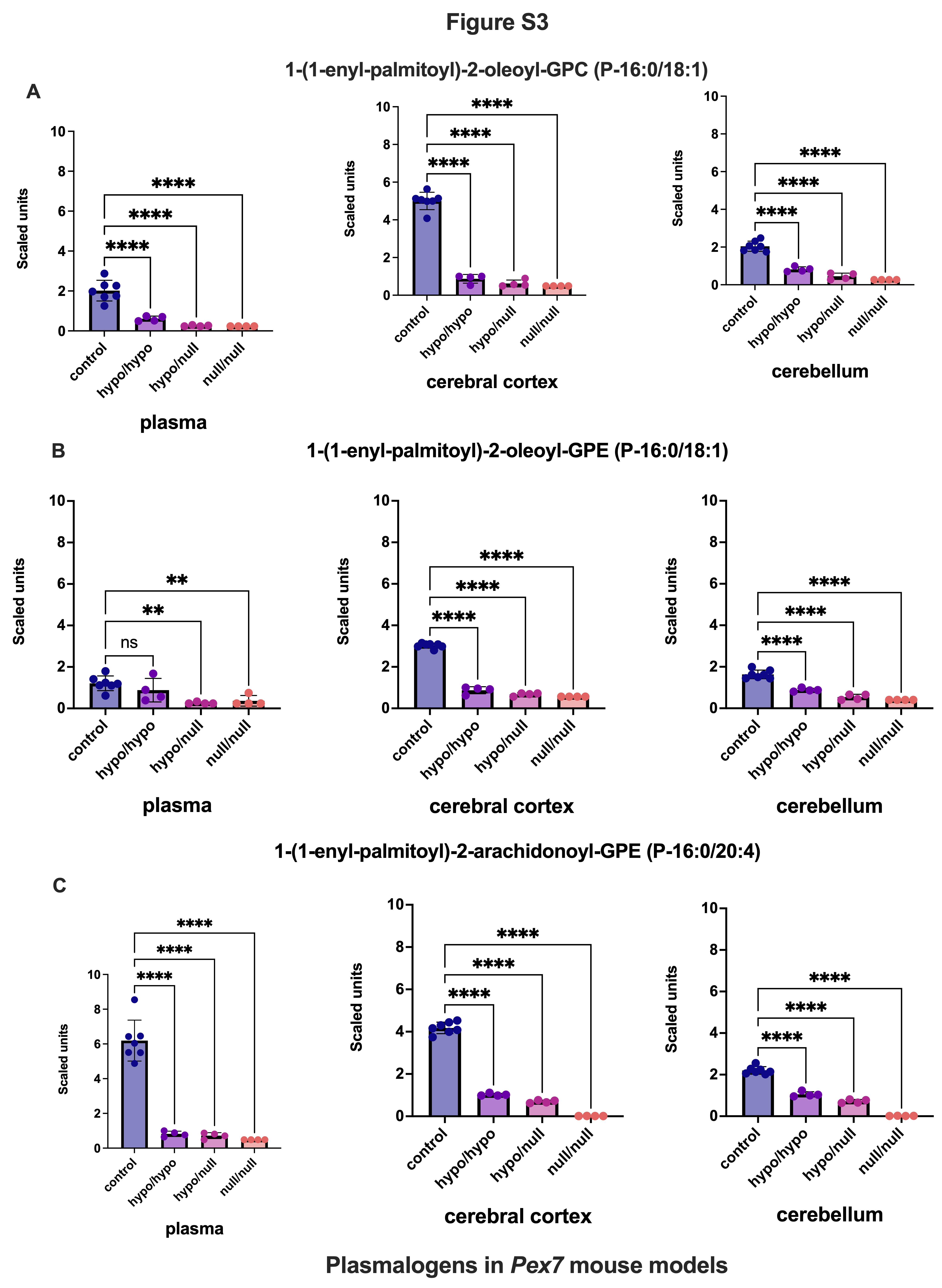

Supplement: Supplementary file 1 [file biomolecules-16-00006-s001.zip › FigureS3 plasmalogen.tiff]

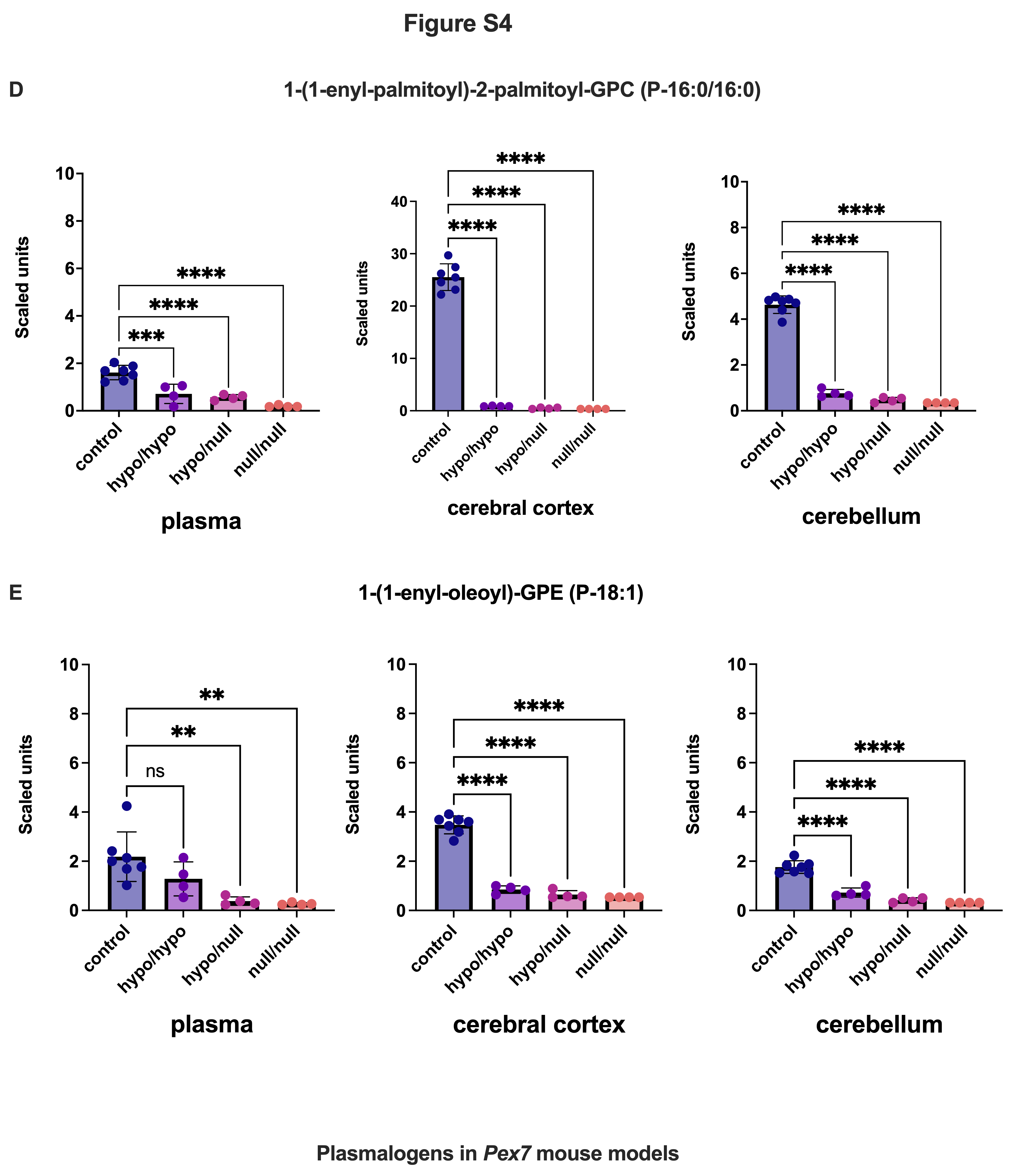

Supplement: Supplementary file 1 [file biomolecules-16-00006-s001.zip › FigureS4 plasmalogen.tiff]

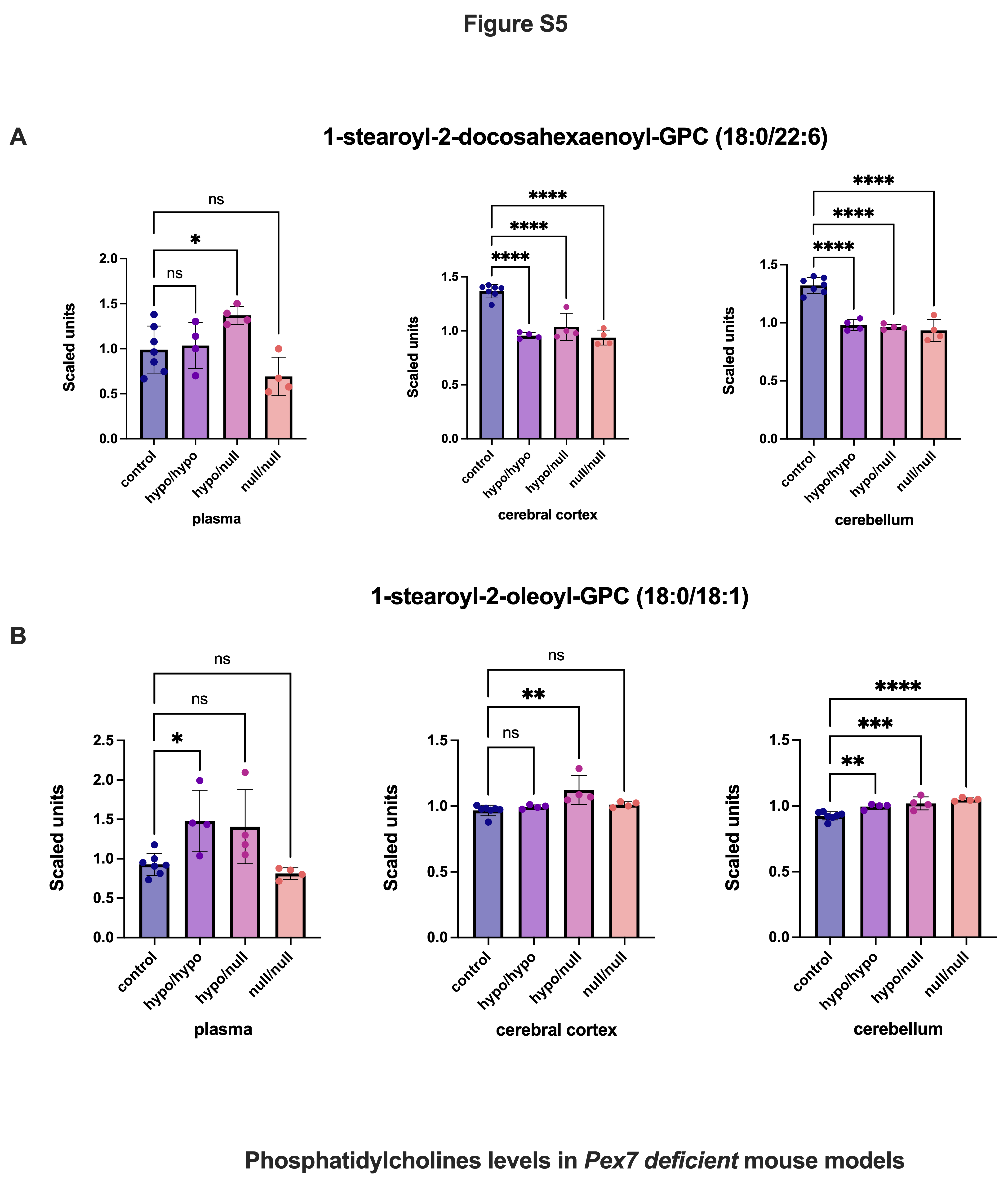

Supplement: Supplementary file 1 [file biomolecules-16-00006-s001.zip › FigureS5 PC.tiff]

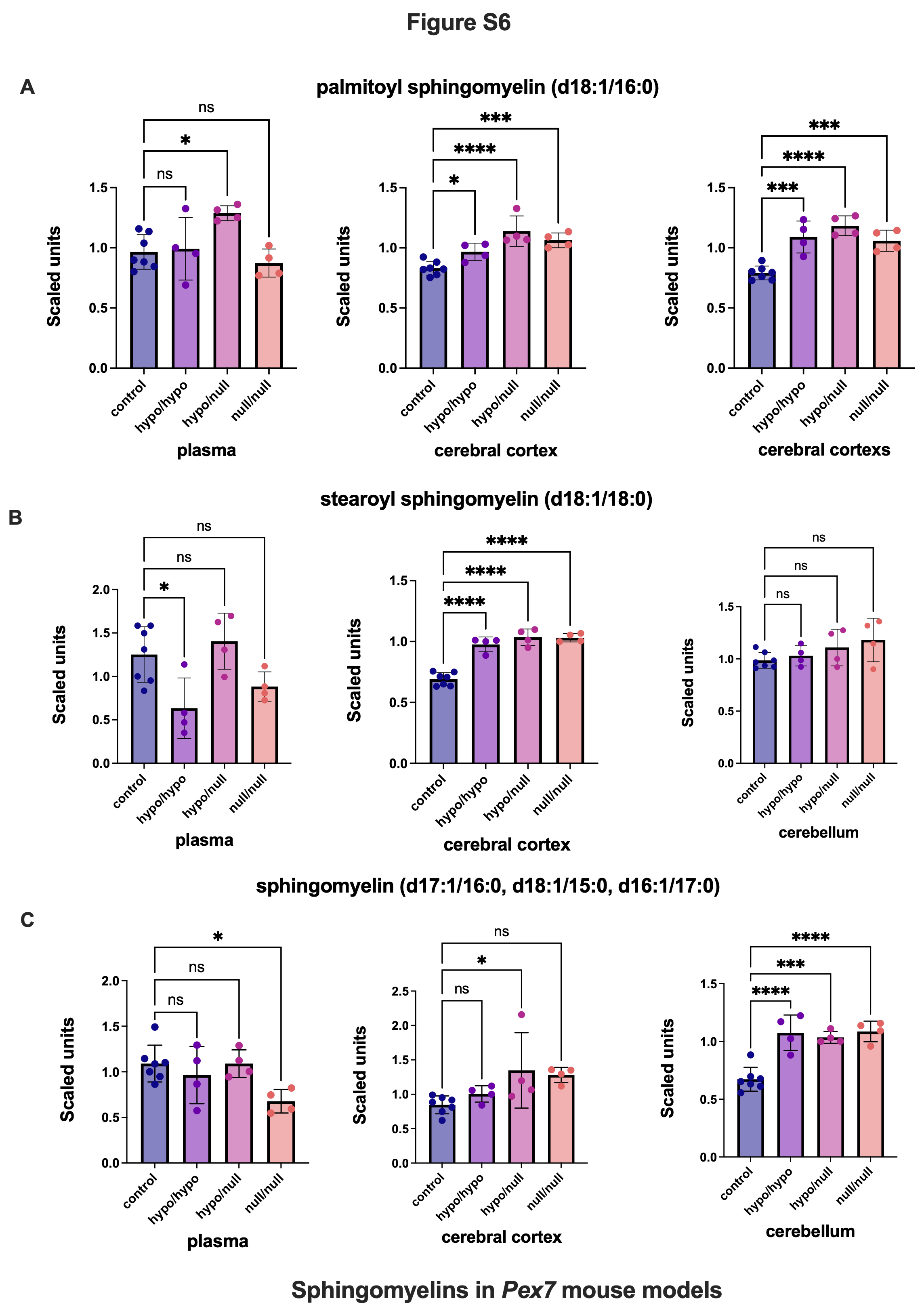

Supplement: Supplementary file 1 [file biomolecules-16-00006-s001.zip › FigureS6 Sphingomyelins.tiff]

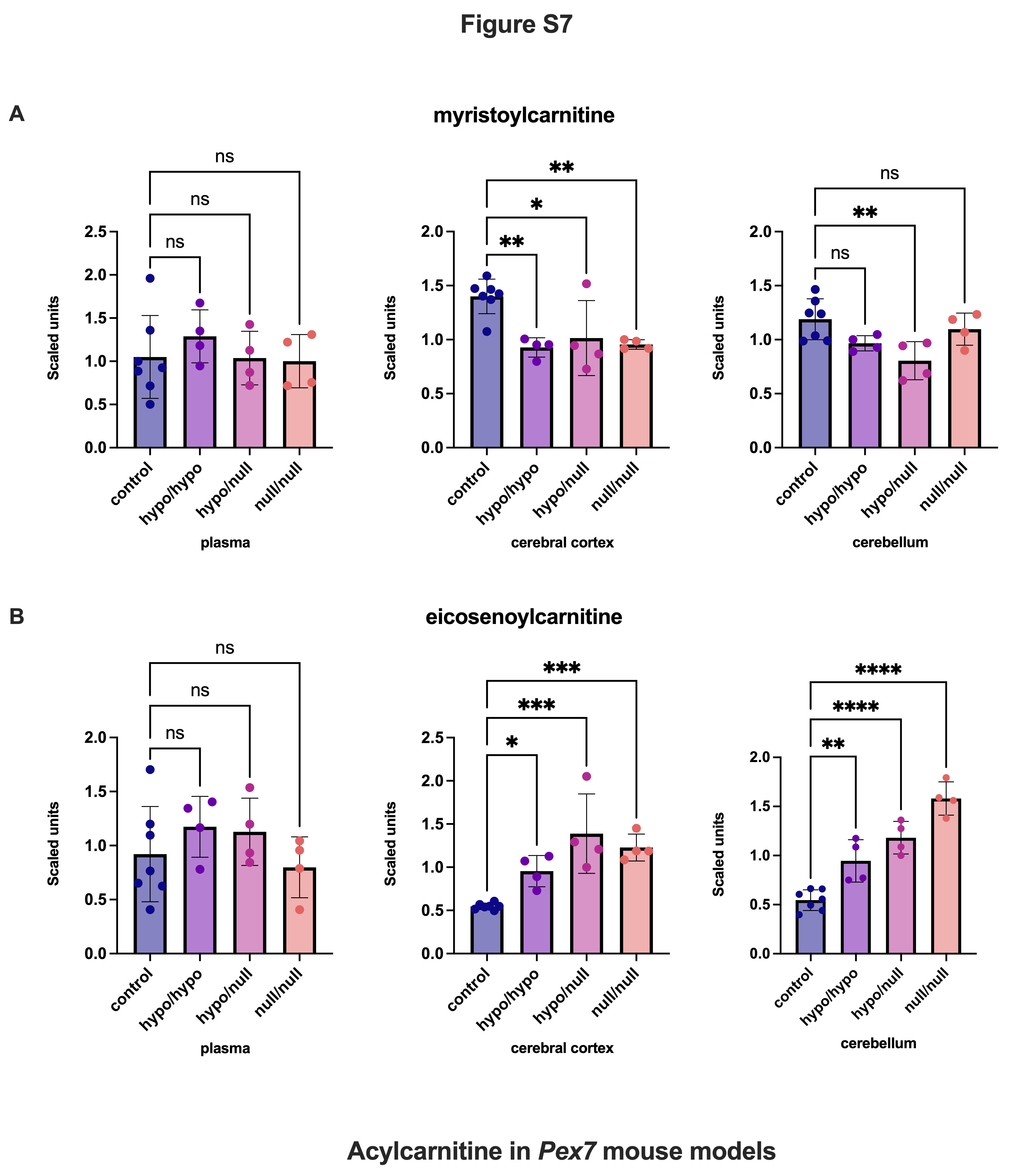

Supplement: Supplementary file 1 [file biomolecules-16-00006-s001.zip › FigureS7 Acylcarnitine.tiff]

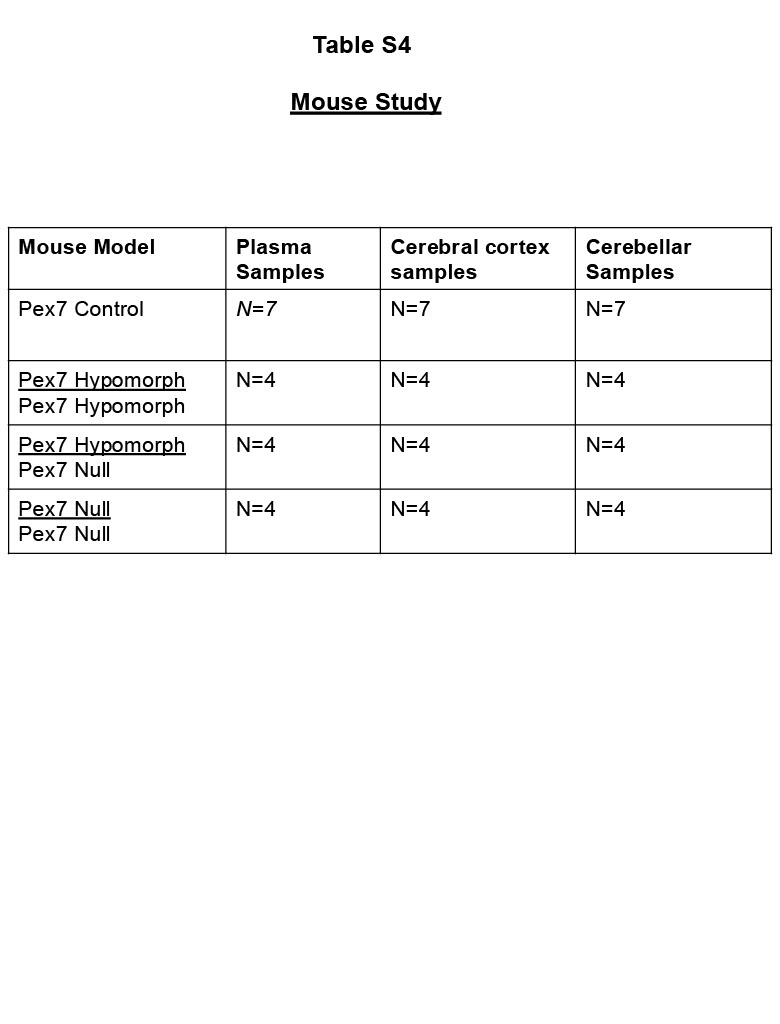

Supplement: Supplementary file 1 [file biomolecules-16-00006-s001.zip › Table S4.jpg]
